# Bifunctional nanoprobe for simultaneous detection of intracellular reactive oxygen species and temperature in single cells

**DOI:** 10.1038/s41378-024-00814-1

**Published:** 2024-11-19

**Authors:** Yanmei Ma, Weikang Hu, Jian Hu, Muyang Ruan, Jie Hu, Ming Yang, Yi Zhang, Hanhan Xie, Chengzhi Hu

**Affiliations:** 1 Shenzhen Key Laboratory of Biomimetic Robotics and Intelligent Systems, Department of Mechanical and Energy Engineering, Southern University of Science and Technology, Shenzhen, 518000 China; 2 Department of Electrical and Electronic Engineering, Southern University of Science and Technology, Shenzhen, 518000 China

**Keywords:** Nanosensors, Biosensors

## Abstract

Living cells can rapidly adjust their metabolic activities in response to external stimuli, leading to fluctuations in intracellular temperature and reactive oxygen species (ROS) levels. Monitoring these parameters is essential for understanding cellular metabolism, particularly during dynamic biological processes. In this study, we present a bifunctional nanoprobe capable of simultaneous measurement of ROS levels and temperature within single cells. The nanoprobe features two individually addressable nanoelectrodes, with platinum (Pt) and nickel (Ni) coatings on both sides. At the tip, these two metal layers form a nano-thermocouple, enabling precise intracellular temperature measurements, while the Pt layer facilitates selective ROS detection. This dual functionality allows for real-time monitoring of cellular responses during synergistic chemo-photothermal therapy of cancer cells and zebrafish embryos subjected to mitochondrial toxic stress. Our results demonstrate that the nanoprobe effectively measures increases in temperature and ROS levels in HeLa cells undergoing chemo-photothermal therapy, as well as in chemically stimulated zebrafish embryos. By providing detailed analysis of submicrometer-scale temperature and ROS variations within living cells, this nanoprobe offers valuable insights into cellular processes and holds promise for early disease detection and drug development.

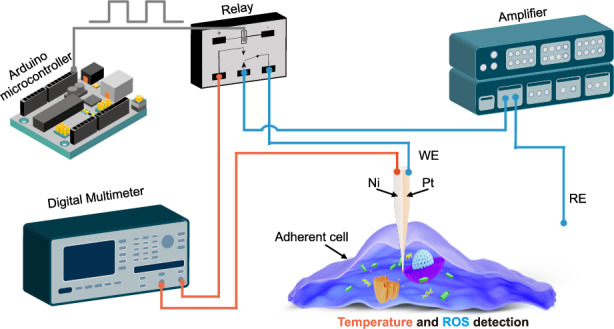

## Introduction

Probing intracellular dynamics is fundamental for uncovering cellular functions and physiological activities. Cells are the site of numerous biological processes and reactions. Their intracellular biophysical and biochemical properties dominate the operation and interaction of cells, as well as behavior or response to various stimuli. Over the decades, significant efforts have been made to develop nanotechnology-based tools for enabling subcellular interrogation with adequate sensitivity and specificity^1,2^. Nanotools and techniques, such as nanoparticles^3^, nanowires^4^, nanotubes^5^, modified atomic force microscopy probes^6^, fluorescent proteins, and molecules^7^, have been employed to investigate intracellular activity and access intracellular domains. These versatile nanodevices allow for the precise measurement of mechanical and electrical characteristics^8–11^, pH levels^12^, concentrations of various ions and molecules^13–15^, and protein dynamics under normal and pathological conditions^16–18^, which greatly enhance our understanding of cellular processes.

Intracellular reactive oxygen species (ROS), including superoxide anion radical (O_2_·^−^), hydroxyl radical (·OH), hydrogen peroxide (H_2_O_2_), and singlet oxygen (^1^O_2_), are by-products of cellular respiration, acting as both damaging agents and signaling molecules. ROS can destroy cell structures by oxidizing or inactivating DNA, proteins, lipids, and other biomolecules, leading to processes such as apoptosis, necrosis, or autophagy, particularly in cancer cells^19–21^. At the same time, ROS are crucial in signaling pathways related to growth, differentiation, and stress responses^22^. Enabled by nanoelectrochemical biosensors, the spatiotemporal characteristics of continuous ROS leakage have been precisely determined during frustrated phagocytosis of high-aspect-ratio glass nanofibers in single macrophages^23^. Similarly, temperature is another critical factor that influences cellular function and development. The local temperature within live cells directly impacts the rate of enzymatic reactions, protein folding, molecular composition, and cell volume expansion^24,25^. Both intracellular temperature and ROS have been considered key modulators that collectively sustain the essential biological activities within cells^26^. For example, cell temperature and ROS are both associated with mitochondrial oxidative phosphorylation and cytoplasmic glycolysis^27^. Effective cancer treatments often trigger synergistic increases in ROS production and elevated temperatures in cancer cells^28,29^. Thus, monitoring the interplay between intracellular temperature and ROS levels is essential for identifying the signaling cascades and treatment mechanisms, as well as for assessing treatment efficacy, which is ultimately constructive for coming up with more personalized and effective treatment plans for patients.

**Fig. 1:**
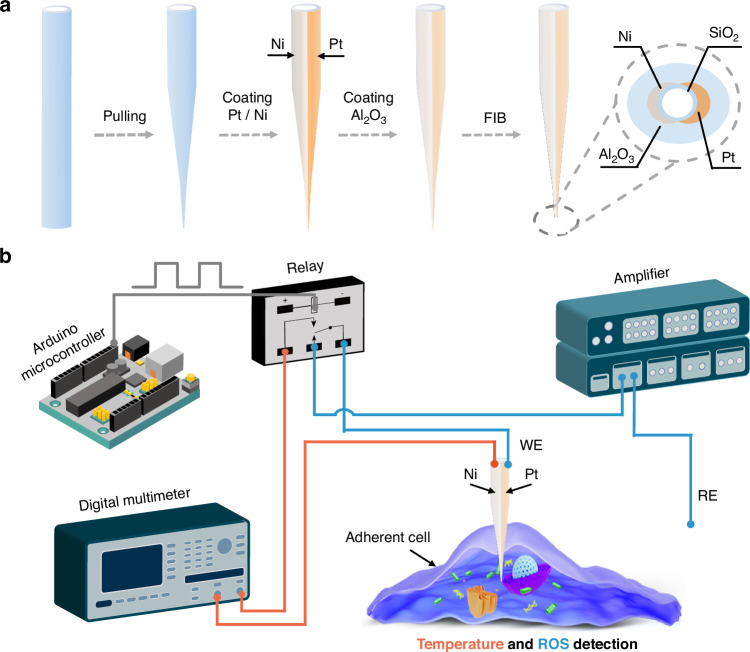
Fabrication process of the nanoprobe and schematic diagram of programmed intracellular ROS and temperature detection. **a** The fabrication process involves pulling the capillary to create a nanometer-sized tip, coating the glass nanopipette with Pt and Ni, and insulating the Pt and Ni layer with Al_2_O_3_. The insulating layer at the tip is then milled using FIB to expose the Pt and Ni. **b** Schematic illustrating the electrochemical detection of ROS and temperature by the nanoprobe in a living cell. The relay, programmed by the Arduino microcontroller, switches between ROS and temperature detection. When the nanoprobe’s Pt and Ni terminals are connected to a multimeter, it measures intracellular temperature. When the Pt terminal is connected to an amplifier with a reference electrode (RE) in the culture medium, it detects intracellular ROS

Nanoprobes, which have nanometer-sized tips, can be directly inserted into living cells to probe the intracellular environment with minimal disruption to cellular activity. While various methods have been developed to sense intracellular ROS and temperature separately, achieving simultaneous measurement with high sensitivity, accuracy, and selectivity using a nanoprobe presents significant challenges due to size constraints. In this work, we developed a bifunctional nanoprobe capable of simultaneously detecting intracellular ROS levels and temperature. The nanoprobe was fabricated by coating both sides of a nanopipette with Pt and Ni (Fig. 1a). The Pt and Ni layers at the tip were bridged by controlling the coating layer thickness, forming a nano-thermocouple that detects the intracellular temperature^30^. The exposed Pt layer can be selectively used to sense ROS in living cells. Each sensing unit exhibited excellent selectivity for H_2_O_2_ (used as a representative of ROS) or temperature. The nanoprobe was utilized to monitor the variations of ROS and temperature during the synergistic chemo-photothermal therapy against HeLa cells using a 2D ultrathin MXene-based drug-delivery nanoplatform (Ti_3_C_2_@DOX). A significant increase in intracellular ROS and temperature was observed, with temperature rise correlating with the concentration of Ti_3_C_2_@DOX. Additionally, the nanoprobe was used to study the changes of ROS and temperature in zebrafish embryos upon exposure to the mitochondrial uncoupler drug carbonyl cyanide p-trifluoromethoxy-phenylhydrazone (FCCP). The FCCP-treated group showed a marked increase in signal compared to untreated zebrafish embryos, indicating ROS production and temperature elevation induced by mitochondrial stress. Our work demonstrates a versatile nanoprobe for effective monitoring of intracellular ROS and temperature, with potential applications in early disease detection and drug development.

## Results and discussion

### Fabrication and characterization of the nanoprobe

The fabrication process of the nanoprobe is depicted in Fig. 1a. Initially, a nanopipette was sputtered with Pt and Ni on both sides, followed by a coating with Al_2_O_3_. The insulation at the tip was then removed using the focused ion beam (FIB). The resulting nanoprobe has three distinct layers in the cross-section of the FIB-cut tip: an inner layer of SiO_2_ from the original nanopipette, a middle layer of Pt and Ni (each 100 nm thick), and an outer layer of Al_2_O_3_ (100 nm thick). A 5 nm of Ti was coated before sputtering Pt and Ni to promote their adhesion to the glass nanopipette. The Al_2_O_3_ layer was selected as an insulation layer due to its excellent thermal and chemical stability. When the Pt layer is connected to a low-noise amplifier (Axopatch 700B), the nanoprobe functions as a ROS sensor, as depicted in Fig. 1b (blue line). Since the Ni and Pt are connected at the tip, these two dissimilar metals form a nano-thermocouple due to the Seebeck effect. Consequently, a voltage is generated when the tip experiences a temperature difference relative to the environment, as depicted in Fig. 1b (orange line). Pt and Ni were selected due to their relatively large difference in Seebeck coefficients, with Pt serving as the common sensing electrode for ROS^31^. This measured voltage can be directly correlated to temperature. In the experiment, ROS detection and temperature measurement were selectively controlled using a single-pole-double-throw signal switching relay (G6EU-134P-US, Omron, Japan), which is programmed by an Arduino microcontroller (Uno R3, Italy). The nanopipette operation was carried out using an automated micromanipulation system previously developed in our lab (Fig. S1)^32^. The system can perform image-based detection of both the cell and nanoprobe tip, as well as precise three-dimensional positioning of the nanopipette tip in proximity to the cell of interest, cell approaching, proximity detection between the nanopipette tip and the cell surface, and cell penetration, with a resolution of 50 nm.

**Fig. 2:**
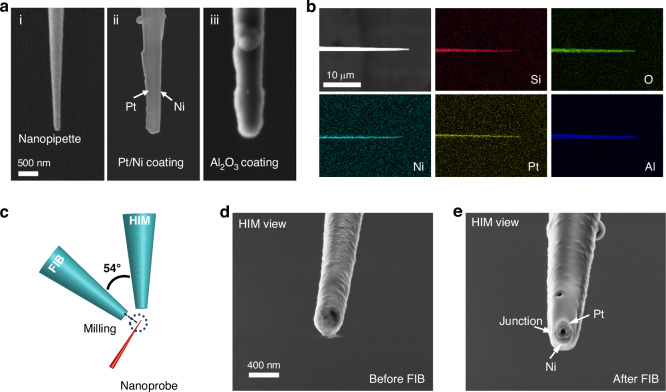
Morphology characterization of the nanoprobe. **a** SEM image showing (i) the glass nanopipette, (ii) the nanopipette coated with Pt and Ni on both sides, and (iii) the nanoprobe coated with Al_2_O_3_. **b** EDS maps of the nanoprobe revealing the presence of Si, O, Ni, Pt, and Al at the nanoprobe tip. **c** Illustration of the FIB milling and subsequent HIM imaging. The nanoprobe sample was positioned perpendicular to the FIB detector, while the HIM detector was at a 54° angle relative to the FIB detector. **d** HIM images of the nanoprobe before FIB cutting and (**e**) after FIB cutting

Each step of the nanoprobe fabrication process was characterized, as shown in Fig. 2a. Initially, the nanoprobe tip had a diameter of approximately 100 nm after the pulling process (Fig. 2a i). After sputtering the Pt and Ni layers, the outer diameter of the nanopipette increased to around 300 nm, and the Pt and Ni layers were connected at the tip of the nanoprobe (Fig. 2a ii). Following the coating of an Al_2_O_3_ insulation layer, the nanoprobe diameter expanded to approximately 500 nm (Fig. 2a iii). Energy-dispersive X-ray spectroscopy (EDS) analysis (Fig. 2b) confirmed that the Pt and Ni layers were continuous and uniformly adhered to the nanopipette. The Pt and Ni layers at the tip were exposed using a FIB coupled with a Helium Ion Microscope (HIM) setup (Fig. 2c). The morphology of the nanoprobe tip before and after FIB cutting is shown in Fig. 2d, e, and Fig. S2. The two metal layers were bridged at the tip, forming a nano-thermocouple capable of detecting intracellular temperature with high precision. The small size of the nanoprobe enables high spatial resolution measurements. Meanwhile, it significantly reduces potential cell damage and supports long-term intracellular analysis.

### Electrochemical response of the nanoprobe

**Fig. 3:**
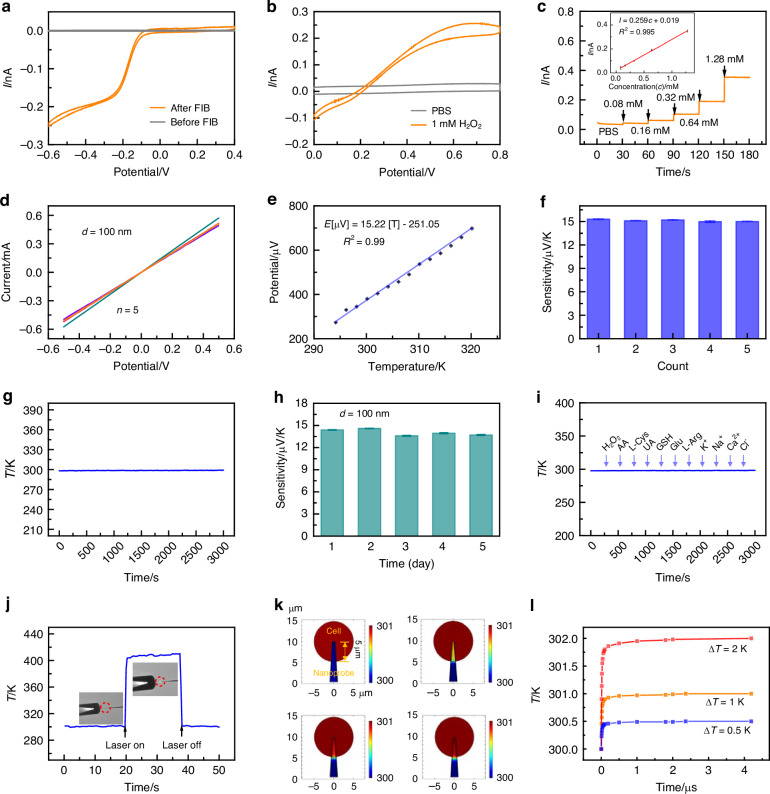
Performance characterization of the nanoprobes. **a** Cyclic voltammogram of the nanoprobes in 1 mM Ru(NH_3_)_6_Cl_3_/1 M KCl solution before and after FIB milling. Scan rate = 100 mV s^−1^. **b** Cyclic voltammograms of the nanoprobe in 1 mM H_2_O_2_ solution and PBS solution. Scan rate = 100 mV s^−1^. **c** Current response to varying H_2_O_2_ concentrations, with the corresponding calibration curve (inset). **d** *I-V* curves of the five individually prepared nanoprobes sputtered with a 100 nm layer of Pt and Ni in the range of -0.5 to 0.5 V in air. **e** Linear voltage dependence on temperature for the nanoprobe in 10 mM PBS, within the range of 294.15–320.15 K. **f** Thermoelectric sensitivity of five individually prepared nanoprobes. **g** Stability of the nanoprobe at 298 K in 10 mM PBS. **h** Sensitivity of the same nanoprobe over five days. **i** Temperature curves of the nanoprobe in 10 mM PBS with various additives. **j** Temperature variation curve caused by laser heating at the tip. **k** Simulated transient response of the nanoprobe when the cell temperature is 1 K higher than the nanoprobe. **l** Temperature variation of the nanoprobe (5 µm away from the tip) after the nanoprobe enters the cell, with the cell temperature higher than the environment by 0.5 K, 1 K, and 2 K

To characterize the electrochemical performance of the nanoprobe, the tip of the nanoprobe was placed in a 1 M KCl solution containing 1 mM Ru(NH_3_)_6_^3+^ before and after FIB milling. The cyclic voltammogram in Fig. 3a shows a limiting current of approximately 0.25 nA after FIB milling, while no obvious current was observed before milling, indicating the steady effectiveness of the insulation layer. The nanoprobe was further characterized in 1 mM H_2_O_2_ and phosphate-buffered solution (PBS) solution before intracellular ROS detection. Compared to PBS, an obvious current increase was observed in H_2_O_2,_ proving the electrochemical response to H_2_O_2_ due to the Pt layer (Fig. 3b). Chronoamperometry experiments were conducted with an overpotential of 0.85 V (Ag/AgCl) to establish a calibration curve. The results showed that the response current for H_2_O_2_ increased linearly with the target analyte concentrations in the range of 0.08–1.28 mM (Fig. 3c). Further experiments were conducted to evaluate the current readouts in the presence of common disruptors, including various amino acids and ions. Our results demonstrate that the nanoprobes exhibit high selectivity and reliability for ROS detection under conditions that mimic the cell environment (Fig. S3a). Additionally, we found that temperature variations have little impact on ROS detection (Fig. S3b).

### Temperature measurement by the nanoprobe

Due to the different thermoelectric properties of Ni and Pt, the Pt/Ni junction at the nanoprobe’s tip generates a thermoelectric potential in response to temperature variations between the tip (the hot point) and the tail end (the cold point). The hot end functions as the temperature-sensing region, while the cold end serves as the temperature reference. The thermoelectric potential (*E*_T_) across a thermocouple can be determined by the equation:

$${E}_{{\rm{T}}}=\varDelta S({T}_{{\rm{h}}}-{T}_{{\rm{c}}})$$

where *ΔS* denotes the thermoelectric sensitivity, *T*_h_ represents the temperature at the hot end (measured temperature), *T*_c_ indicates the temperature at the cold end (room temperature)^33^.

After coating the Pt and Ni layers on both sides of the nanopipette, the two metals were bridged at the tip of the nanoprobe, as indicated by the *I*-*V* curve in Fig. 3d. The resistance of the nanoprobe was 1.05 kΩ (*n* = 5). We further evaluated the nanoprobe’s sensitivity, repeatability, stability, and responsiveness to thermal changes. A calibration curve for temperature measurement using the nanoprobe was established in 10 mM PBS, as shown in Fig. 3e. As the temperature of the solution increased, the *E*_T_ values increased accordingly. A clear linear relationship was observed in the range of 294.15 K to 320.15 K (20–46 °C), with the equation *E*_[µv]_ = 15.22 T–251.05, *R*^2^ = 0.99. The slope, representing the thermoelectric sensitivity of the sensor, showed excellent consistency and repeatability across five independently prepared nanoprobes, with an average sensitivity of 15.02 µV/K (n = 5) (Fig. 3f). This experimental value is close to the theoretical sensitivity (15 µV/K), which is consistent with the Seebeck coefficient of Ni and Pt^34^.

The stability of the nanoprobe was also evaluated. The *E*_T_ remained nearly constant when the nanoprobe was immersed in a 298 K PBS solution for continuous monitoring for 3000 s (Fig. 3g). The sensitivity of the Pt/Ni nanoprobe showed negligible change over five days, demonstrating its exceptional stability (Fig. 3h). The anti-interference capability of the nanoprobe was also tested by adding various metal ions, ROS, and amino acids to the solution during temperature measurement. As shown in Fig. 3i, no discernible alterations were observed, underscoring the high selectivity of the nanoprobe for temperature. To study the influence of metal layer thickness, a 50 nm coating of both Pt and Ni was applied on the two sides of the nanopipette. The corresponding *I*-*V* curve is shown in Fig. S4a. The resistance of the nanoprobe (d = 50 nm) was 3.86 kΩ (n = 5) (Fig. S4 b). A calibration curve for different temperatures was also established (Fig. S5a), with the correlation of *E*_[µv]_ = 13.95 T–2.45, *R*^2^ = 0.99. The average sensitivity of five independent nanoprobes is 13.38 µV/K (Fig. S5b), which is slightly lower than that of the nanoprobe with a 100 nm layer. This discrepancy could be attributed to the nanosized effects on electron transport in the tip region, where the Seebeck coefficients of thin film material are influenced by both the film thickness and the electron mean free path^35^.

The reversibility of the nanosensor is crucial for real-time, continuous, and accurate monitoring. We observed temperature fluctuations within the range of 298-325 K. As depicted in Fig. S6a, the nanoprobe exhibited a remarkably reversible temperature response as the tip was periodically inserted into hot water (325 K) and retracted into air (298 K). To evaluate the temporal response of the nanoprobe, we monitored the temperature fluctuations caused by laser heating of the tip. As shown in Fig. 3j and Fig S6 b, a rapid temperature response was observed. The slight decrease in measured voltage in Fig S6 b is attributed to the gradual shift in the laser spot’s position relative to the nanoprobe tip, which caused a reduction in the tip’s temperature over time. To investigate the transient heat transport of the nanoprobe in a cytoplasmic environment, we conducted finite element simulations using COMSOL. Detailed descriptions of the modeling and implementation are provided in the Supporting Information (Text S1, Fig. S7). Initially, the temperature of the nanoprobe was set to 300 K, while the cell temperature was 301 K. When the nanoprobe tip was inserted 5 µm into the cell, the simulation showed that the nanoprobe reached the intracellular environment within 3.28 µs (Fig. 3k). We also evaluated the response times for the temperature differences of 0.5 K and 2 K, which were found to be 2.36 µs and 4.19 µs, respectively (Fig. 3l, Fig. S8, Fig. S9).

The development of a bifunctional nanoprobe is crucial due to its ability to provide more comprehensive insights into cellular function while minimizing cell damage. Conventional approaches often require multiple separate nanoprobes or measurements, which can disrupt the delicate cellular structure and interfere with the very processes being studied^36^. This can lead to inaccuracies, particularly when it is difficult to ensure that different probes are measuring the same intracellular location. The bifunctional nanoprobe reduces the damage to the cell caused by insertion operations. A nanoprobe integrated with both functions only requires a single insertion to obtain both signals, whereas using two separate nanoprobes would need two insertions. Although it has been reported that the penetration of nanoscale tips into cells is minimally invasive, repeated penetrations can still affect cell viability and function^37^. Therefore, while obtaining multiple types of intracellular information, it is important to minimize the interference caused by the measurements as much as possible. Besides, simultaneous detection improves spatial positioning accuracy. Although cells are small in volume, their intracellular environment is highly complex. Even for the same biomolecule, such as ROS or Ca²^+^, or physical factors like temperature, there can be significant differences in different regions within the cell^22,38,39^. Therefore, to obtain two or more signals from the same region, the nanoprobe would need to be repeatedly inserted into the same location within the cell. However, repeated operations make it difficult to ensure consistency in the insertion position, leading to inaccurate signals.

### Intracellular sensing under synergistic photothermal-/chemotherapy

Cancer cells typically exhibit higher ROS levels compared to normal cells due to increased metabolic activity and mitochondrial dysfunction. Cancer therapies, including radiation, immunotherapy, and certain chemotherapeutic agents, can further escalate ROS levels within cancer cells^40^. Monitoring intracellular temperature and ROS concentration is crucial for optimizing cancer therapies^41^. Strategies that modulate these factors, such as combining hyperthermia with ROS-inducing treatments, are actively studied to enhance cancer therapy^42,43^.

**Fig. 4:**
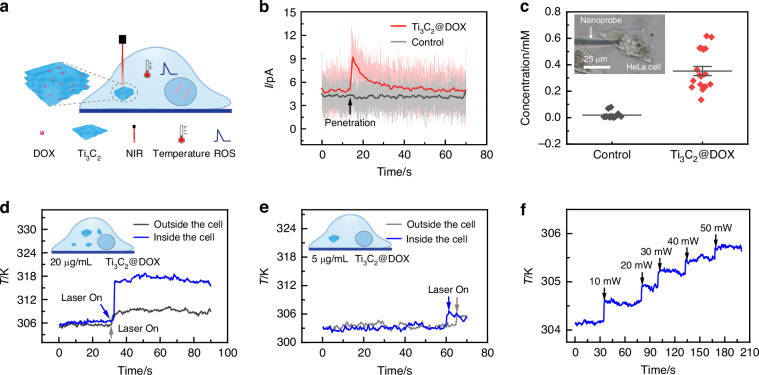
Real-time measurement of ROS and temperature in adherent HeLa cells during photothermal-chemotherapy treatment. **a** Schematic illustration of intracellular temperature and ROS detection, Ti_3_C_2_@DOX was endocytosed by HeLa cells. Under NIR irradiation, both intracellular temperature and ROS concentration increased, as detected by the nanoprobe. **b** Intracellular current traces measured by the nanoprobe in HeLa cells with Ti_3_C_2_@DOX (orange) and without Ti_3_C_2_@DOX (gray). **c** Quantification of intracellular ROS concentrations in 19 individual cells (inset shows the nanoprobe inserted into a Ti_3_C_2_@DOX-treated HeLa cell). **d** Thermoelectric potential traces collected from the nanoprobe when penetrating (blue) and positioned near (gray) a single HeLa cell after treatment with 20 µg/mL Ti_3_C_2_@DOX and NIR irradiation. **e** Thermoelectric potential traces collected from the nanoprobe during cell penetration (blue) and when positioned near (gray) a single HeLa cell after treatment with a lower concentration of 5 µg/mL Ti_3_C_2_@DOX and exposure to NIR irradiation. **f** Thermoelectric potential traces of a single HeLa cell treated with Ti_3_C_2_@DOX under different NIR intensities

Ti_3_C_2_ MXene, a novel two-dimensional material, exhibits excellent biocompatibility and photothermal effects under NIR light. Using DOX as a model drug, Ti_3_C_2_@DOX shows high drug loading capacity and pH-/thermo-sensitivity, where drug release of the nanosystem can be promoted in an acidic tumor microenvironment or by elevated temperature under 980 nm laser irradiation^44^. Here, we studied changes in intracellular temperature and ROS during the photothermal-/chemotherapy of cancer cells using a MXene-based therapeutic nanosystem, as illustrated in Fig. 4a. HeLa cells were incubated with Ti_3_C_2_@DOX nanocomposites at a concentration of 20 µg/mL for 10 hours. After incubation, extracellular Ti_3_C_2_@DOX was removed by washing with PBS. The nanoprobe was robotically inserted into the cell. Upon cellular penetration, a significant and immediate increase in the electrochemical current was observed, as shown in Fig. 4b. This spike indicates the successful interaction of the nanoprobe with the intracellular environment. In contrast, the control group which was not treated with Ti_3_C_2_@DOX (Fig S10), exhibited negligible ROS levels, highlighting the effectiveness of Ti_3_C_2_@DOX in elevating ROS concentrations within cancer cells. These results also indicate that the detected signals arise from the effects of the chemical agents, rather than mechanical stimulation caused by the insertion. Additionally, the experiment was extended to measure ROS levels in 19 cells. The ROS concentration in the cells was determined based on Faraday law *n* = *Q/ZF*, where *n* is the amount of substance, *Q* is the amount of charge (obtained by integrating the current over time), *F* is the Faraday constant (*F* = 96485 C/mol), and *Z* is the number of electrons transferred (in this case, *Z* = 1). Given the average diameter of HeLa cells is approximately 10 µm, we were able to calculate the corresponding ROS concentration in the cells. The results revealed that Ti_3_C_2_@DOX treatment consistently increased intracellular ROS concentration to approximately 0.44 mM, as depicted in Fig. 4c. These findings provide evidence for the ROS-inducing capability of Ti_3_C_2_@DOX and its potential as an effective chemotherapeutic agent.

As shown in Fig. 4c, the insertion of the nanoprobes into cells does not show significant influence to the detected signals. However, the physical insertion of nanoprobes into cells can still potentially induce a mechanical response. This mechanical stimulation, caused by the deformation or disruption of the cell membrane, could trigger various cellular responses, such as ion channel activation, changes in membrane tension, or cytoskeletal reorganization^45^. In general, several strategies can be employed to decouple the effects of mechanical stimulation from chemical stimulation: 1) Performing control experiments where the nanoprobes are inserted without introducing any chemical stimuli allows for the characterization of the mechanical response alone; 2) Use of mechanically unresponsive cells (genetic knockouts of key mechanotransduction proteins, e.g., integrins, ion channels like PIEZO1^46^); 3) Temporal analysis of signals: Mechanical responses are often immediate, while chemical responses, particularly intracellular signaling cascades, may take longer to manifest^47^.

The intracellular temperature was monitored during photothermal therapy. A 980 nm laser with a power of 10 mW was then used to irradiate the cell, ensuring that the laser beam did not directly impact the nanoprobe tip. Upon activation of the laser, a significant increase in thermoelectric voltage was observed, corresponding to a temperature rise of approximately 10 K. In contrast, when the probe was positioned outside the cell under the same experimental conditions, the temperature increase was much smaller, around 3 K (Fig. 4d). Additionally, when the concentration of Ti_3_C_2_@DOX was reduced to 5 µg/mL, the intracellular presence of Ti_3_C_2_@DOX was visibly lower compared to the higher concentration group (20 µg/mL), as evidenced by bright-field imaging (Fig. S11). Under these conditions, the cell temperature increased by only about 4 K (Fig. 4e). These findings indicate that the observed increase in cell temperature is directly related to the presence of Ti_3_C_2_@DOX within the cells. Additionally, we examined the temperature changes within the cells under varying laser intensities. As shown in Fig. 4f, there is a clear correlation between laser intensity and cell temperature, with higher laser intensities leading to greater temperature increases. These results provide precise measurements of temperature changes at the single-cell level during photothermal therapy, demonstrating that the temperature rise is primarily due to the amount of photothermal material inside the cells. This multifunctional nanoprobe is capable of accurately measuring both intracellular temperature changes and ROS levels within cancer cells during photothermal and chemotherapy. The ability to monitor these parameters in real-time offers valuable insights into the cellular responses to therapy, facilitating the optimization of treatment protocols and enhancing therapeutic efficacy.

### Intracellular analysis of zebrafish embryos under mitochondrial toxic stress

**Fig. 5:**
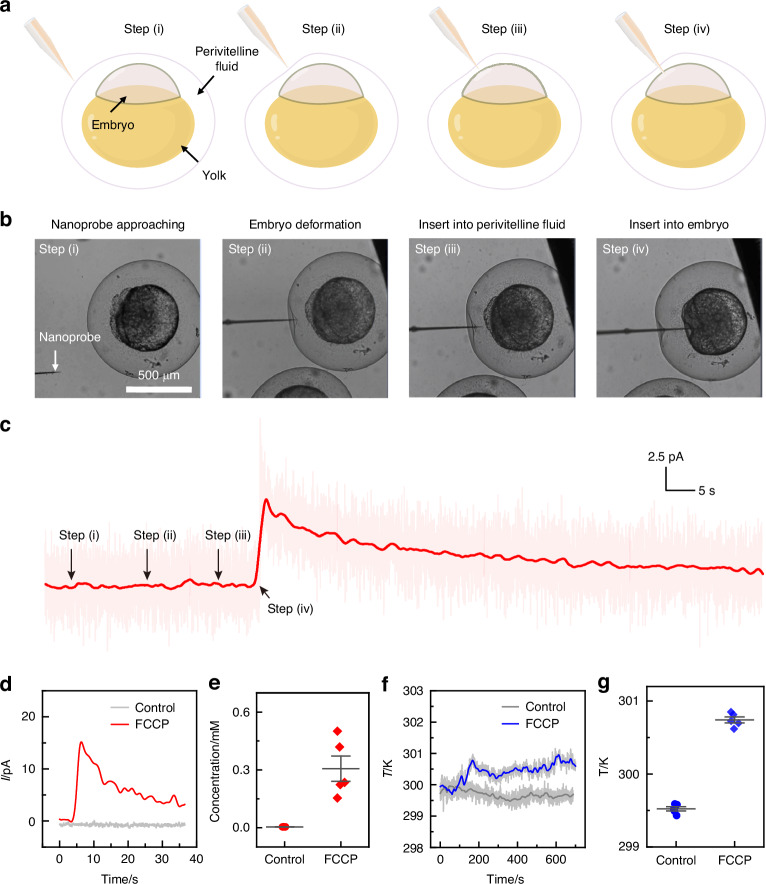
Measurement of ROS and temperature in zebrafish embryos under mitochondrial toxic stress. **a** Schematic illustration of the experimental process for measurement, including steps: (i) nanoprobe approaching the embryo, (ii) embryo deformation, (iii) insertion into the perivitelline fluid, (iv) insertion into the embryo. **b** Corresponding bright-field images of a zebrafish embryo measured at each step in (**a**). **c** Current trace corresponding to ROS detection in a zebrafish embryo measured at each step described in (**a**)**. d** Current traces collected from the nanoprobe after penetrating single zebrafish embryos treated with FCCP and a control group (no FCCP), with an applied potential of 0.85 V. **e** Comparison of ROS concentration in five zebrafish embryos from the FCCP-treated group and the control group. **f** Time variation of temperature traces measured in zebrafish embryos after FCCP treatment and in the control group. **g** Comparison of temperature in five zebrafish embryos from the FCCP-treated group and the control group

We further employed our nanoprobe to investigate mitochondrial respiration and energy metabolism associated with mitochondrial toxicity in zebrafish embryos. FCCP, a potent mitochondrial uncoupler, was utilized to induce mitochondrial damage by disrupting the proton gradient within the mitochondria^48^. The procedure of intracellular sensing is illustrated in Fig. 5a, b. FCCP was added to the zebrafish embryo medium at a concentration of 60 µM to initiate the stimulation of mitochondrial stress (step i). The nanoprobe was robotically maneuvered towards a zebrafish embryo. Notably, no significant current changes were observed when the embryo was gently deformed or when the nanoprobe tip was inserted into the perivitelline fluid areas (step ii and step iii). However, a significant increase in current was detected (at 0.85 V versus Ag/AgCl electrodes) when the nanoprobe tip penetrated the embryo (step iv), as demonstrated in Fig. 5c (red curve). This substantial current increase signifies the successful detection of elevated ROS levels within the FCCP-treated zebrafish embryos. In a control experiment where no FCCP was added, no significant current response indicative of increased ROS levels was recorded in individual zebrafish embryos (Fig. 5d, gray curve). Statistical analysis was performed using five FCCP-treated embryos. As depicted in Fig. 5e, the FCCP-treated group exhibited a noticeable signal increase compared to untreated zebrafish embryos, confirming the mitochondrial stress-induced ROS production. These results validate the capability of our nanoprobe to quantitatively monitor intracellular ROS in real-time, even in complex, three-dimensional biological systems such as zebrafish embryos.

Sequentially, the nanoprobe was electrically switched to monitor intracellular temperature, and the resulting thermoelectric potentials were recorded. As depicted in Fig. 5f, a significant elevation in intracellular temperature can be found in FCCP-treated embryos, whereas no notable change was detected in the control group. The entire process of intracellular temperature change was continuously monitored. It was found that the intracellular thermal signal increased within 3 minutes of FCCP addition and remained elevated for more than 10 minutes. Similar experiments were conducted on another five FCCP-treated embryos. Compared to the control group, the average temperature increase of the FCCP-treated group was approximately 1.2 K, as shown in Fig. 5g. Compared to the fluorescent nanogel-based approach for detecting intracellular temperature, the nanoprobe-based method developed in this study offers superior accuracy and enhanced temporal resolution in detecting intracellular thermal signals. By integrating with a high-precision robotic nanomanipulation system, high spatiotemporal intracellular sensing in living cells can be achieved. These advancements highlight the nanoprobe’s potential for detailed and dynamic intracellular analysis, paving the way for more precise and insightful studies of cellular responses under various physiological and pathological conditions.

## Conclusion

In summary, this study has successfully developed an innovative bifunctional nanoprobe capable of real-time monitoring of intracellular ROS and temperature. The nanoprobe, with a precisely engineered tip size of approximately 500 nm, demonstrates remarkable sensitivity, detecting temperature variations from 20 to 46 °C (294.15 K to 320.15 K) and ROS levels from 0.08 to 1.28 mM. The nanoprobe exhibits exceptional stability, selectivity, and response time, which are crucial for accurate and reliable intracellular measurements, particularly in dynamic cellular environments. In the study of photothermal and chemotherapy on HeLa cells, the ROS concentration within the cells was observed to reach up to 0.4 mM during treatment. Notably, a positive correlation was found between the elevation of cellular temperature and the concentration of photothermal materials within the cells, offering valuable insights into the mechanisms of these therapeutic approaches. Further experiments conducted on zebrafish embryos demonstrated the nanoprobe’s effectiveness in monitoring fluctuations in intracellular temperature and ROS levels induced by chemical stimuli, specifically FCCP. The FCCP-treated group showed a marked increase in signal compared to untreated zebrafish embryos, indicating ROS production and temperature elevation induced by mitochondrial stress. This bifunctional nanoprobe provides real-time, simultaneous measurements of two critical cellular parameters, potentially enhancing disease diagnosis and treatment strategies by providing more accurate and comprehensive data on cellular responses to various stimuli and therapeutic interventions.

## Materials and methods

### Materials and reagents

H_2_O_2_ was purchased from Aladdin Reagent Co., Ltd. (Shanghai, China). FCCP was purchased from GLPBIO. The physiological PBS solution (10 mM, pH 7.4) containing 137 mM NaCl, 2.7 mM KCl, 10 mM Na_2_HPO_4_, 2 mM KH_2_PO_4_ was obtained from Solarbio. Ru(NH_3_)_6_Cl_3_ was purchased from Sigma-Aldrich (USA). Zebrafish embryos were provided by Professor Dong Liu from the Department of Life Science at the Southern University of Science and Technology. Ti_3_C_2_@DOX was synthesized following our previously established protocols^49^. Borosilicate glass capillaries (BF-100-50-10), were acquired from Sutter Instrument Co. The CO_2_-based laser puller (P-2000 laser puller, Sutter Instrument) was made available by Professor Yi Li from the Department of Microelectronics at Southern University of Science and Technology. All electrolyte solutions used in this study were prepared using ultrapure water with a resistivity of 18.2 mΩ cm.

### Fabrication of the nanoprobes

The nanoprobes were fabricated from borosilicate glass capillaries through a four-step process. The glass capillaries were first subjected to a plasma cleaning for 5 min to remove any surface contaminations. The capillaries were then pulled into nanopipettes with tip diameters of approximately 100 nm using a P-2000 laser puller. The specific parameters for the pulling process were as follows: HEAT: 325; FIL: 3; VEL:30; DEL: 250; and PUL: 150. In the third step, a 5 nm Ti adhesion layer was sputtered onto both sides of the nanopipette by rotating the nanopipette 180° around its axis, using an electron beam evaporator (HHV, TF500, India). Subsequently, 100 nm layers of Pt and Ni were coated onto both sides of the nanopipette, ensuring that the tip of the nanopipette was effectively bridged. This dual-metal coating is crucial for the nanoprobe’s functionality as a nano-thermocouple. Finally, a 100 nm layer of Al_2_O_3_ was deposited over the entire nanopipette using atomic layer deposition (ALD), with a deposition rate of approximately 0.1 nm per cycle. This Al_2_O_3_ layer serves as an insulation barrier, enhancing the probe’s durability and performance. The tip of the nanopipette was then precisely milled with focused ion beam helium ion microscopy (FIB-HIM, Orion Nanofab) to expose the functional tip of the nanoprobe.

### Characterization of the nanoprobes

The morphological characteristics and elemental distribution of the nanoprobe were analyzed using a field-emission scanning electron microscope (FE-SEM, ZEISS Merlin) equipped with an energy-dispersive spectrometer (EDS, Octane Pro) at an acceleration voltage of 5 kV. All bright field images were captured with an inverted fluorescence microscope (Axio Observer 7, Zeiss, Germany).

All extracellular current measurements were conducted using electrochemical stations: the CHI 760E (CHI Instrumental, Inc., USA) and the Autolab PGSTAT302N (Metrohm Autolab, Netherlands). A copper wire was carefully attached to the Pt side of the nanoprobe and connected to the electrochemical station. The electrochemical performance of the nanoprobe was characterized using cyclic voltammetry in a solution of 1 mM Ru(NH_3_)_6_^3+^/1 M KCl before and after FIB milling. The scanning potential was in the range of −0.6 to 0.4 V (versus Ag/AgCl), with a scan rate of 100 mV/s. To evaluate the nanoprobe’s performance in detecting ROS, 1 mM H_2_O_2_ and PBS solutions were employed, with the scanning potential ranging from 0 to 0.8 V at a scan rate of 100 mV/s. Quantitative measurements of various H_2_O_2_ concentrations were performed using chronoamperometry at a fixed potential of 0.85 V.

Thermoelectric signals from the nanoprobes were recorded using a KEYSIGHT 34461 A digital multimeter. Two copper wires, attached to opposite sides of the nanoprobe, were connected to the multimeter. Temperature-controlling experiments were performed using a water bath and calibrated by a commercial mercury thermometer. To assess the temperature response time of the nanoprobe, a laser beam with a light spot diameter of 10 µm and a wavelength of 980 nm was used. For the selectivity test, the nanoprobe tip was immersed in an electrochemical cell containing 10 mM PBS (pH 7.4) maintained at a constant temperature. The selectivity of the nanoprobe was evaluated against a panel of potential interferents, including 1 mM H_2_O_2_, ascorbic acid (AA), L-cysteine (L-Cys), Uric Acid (UA), glutathione (GSH), L-arginine(L-Arg), as well as 10 mM glucose (Glu), physiologically relevant concentrations of ions such as Ca^2+^, Na^+^, K^+^, and Cl^−^.

### Cellular uptake of Ti_3_C_2_@DOX

HeLa cells were cultured in Dulbecco’s Modified Eagle Medium (DMEM) supplemented with 10% fetal bovine serum, 100 IU/ml penicillin, and 100 mg/ml streptomycin in a humidified incubator at 37 °C with 5% CO_2_. The cells were seeded on a 3 cm TC-treated dish and incubated overnight. Subsequently, the cells were subjected to Ti_3_C_2_@DOX nanoparticles at different concentrations for at least 10 h, allowing sufficient time for cellular uptake and response. Prior to intracellular measurements, the cells were washed three times with phosphate-buffered saline (PBS) to ensure the complete removal of extracellular Ti_3_C_2_@DOX nanoparticles.

### Intracellular ROS measurement using the nanoprobes

All intracellular current measurements were conducted using an Axopatch 700B low-noise amplifier and an Axon Digidata 1550B low-noise data acquisition system (Molecular Devices, Sunnyvale, CA). An Ag/AgCl wire served as the reference/counter electrode in these experiments. The whole setup was enclosed in a Faraday cage that was properly grounded to minimize electromagnetic interference. Signals were sampled at 1 kHz. The nanoprobe was securely fixed under the microscope using a specialized holder (Axon Instruments, Union City, CA). The motion control system featured two independent micromanipulators (μMp-4, Sensapex, Finland), each offering four degrees of freedom (DOF). These micromanipulators provided a travel range of 20 mm, a motion resolution of 5 nm, and a maximum speed of 5 mm/s for each degree of freedom.

## Supplementary information

Supplemental Material
