# Supplementary material for: Bifunctional nanoprobe for simultaneous detection of intracellular reactive oxygen species and temperature in single cells
Source: Microsyst Nanoeng. 2024 Nov 19;10:171. doi: 10.1038/s41378-024-00814-1 (PMC11577004; doi:10.1038/s41378-024-00814-1)
Supplement: Supplementary file 1 — Supplemental Material [file 41378_2024_814_MOESM1_ESM.docx]

Supplementary Material

**Bifunctional Nanoprobe for Simultaneous Detection of Intracellular Reactive Oxygen Species and Temperature in Single Cells**

Yanmei Ma^1^, Weikang Hu^1^, Jian Hu^1^, Muyang Ruan^1^, Jie Hu^2^, Ming Yang^1^, Yi Zhang^1^ Hanhan Xie^1^ and Chengzhi Hu^1^*

^1^Shenzhen Key Laboratory of Biomimetic Robotics and Intelligent Systems, Department of Mechanical and Energy Engineering, Southern University of Science and Technology, Shenzhen, 518000, China.

^2^Department of Electrical and Electronic Engineering, Southern University of Science and Technology, Shenzhen 518000, China.

*Corresponding author. Email: hucz@sustech.edu.cn

Text S1. Details of numerical simulation

Finite element simulations were conducted using COMSOL Multiphysics (version 5.2) to model the transient thermal responses of the nanoprobe. The “Heat Transfer in Fluids” physics module was employed to analyze the heat transfer processes. A 2D axisymmetric model, informed by SEM observations, was developed and illustrated in Fig. S7. The model consists of four layers, arranged from the symmetrical axis to the outermost layer of the nanoprobe. From left to right, these four layers are: Air (representing the inner cavity of the nanoprobe) with a diameter of 50 nm; Glass with a thickness of 100 nm; Platinum (Pt) with a thickness of 100 nm; Aluminum oxide (Al_2_O_3_) with a thickness of 100 nm. The surrounding cell has a radius of 5 μm, and the nanoprobe is inserted into the cell with a depth of 5 μm. The thermal conductivities of the materials are as follows: Air, 0.0257 W/m·°C; Glass, 1.38 W/m·°C; Pt, 71.6 W/m·°C; and Al₂O₃, 10 W/m·°C. A free tetrahedral mesh was used for the nanoprobe with a maximum element size of 20 nm. The cell was meshed with a free triangular mesh with a minimum element size of 10 nm.


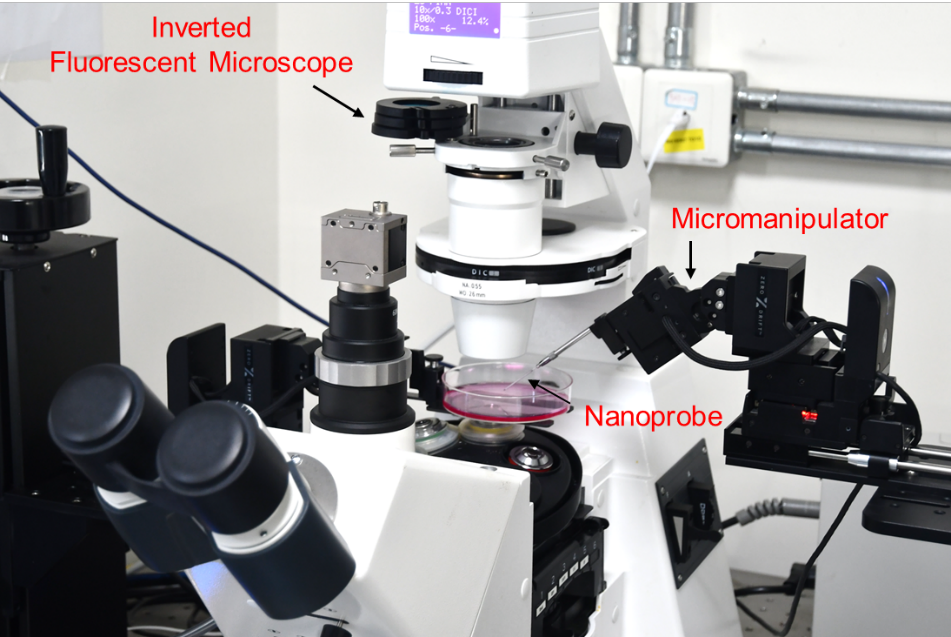


Fig. S1 A photograph image of the automatic micromanipulation system.


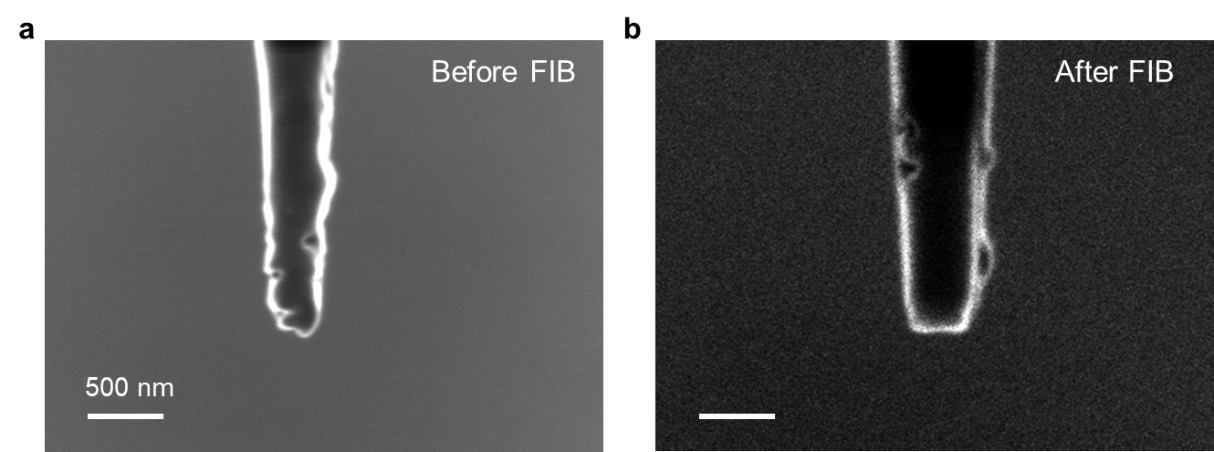


Fig. S2 High-resolution imaging of the nanoprobe before (**a**) and after (**b**) FIB milling, as detected by the FIB detector.


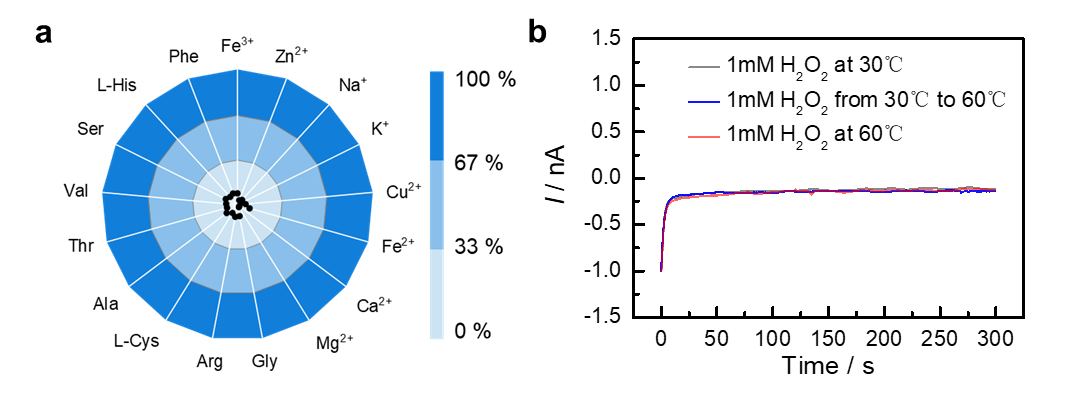


Fig. S3 **a** Selectivity tests of the nanoprobe in the presence of common disruptors such as amino acids and ions. The concentration of H₂O₂ was 1 mM, K⁺ and Na⁺ were each 2 mM, and all other species were 200 µM. **b** Current traces recorded from the nanoprobe in 1 mM H₂O₂ under different temperature conditions: at 30°C (gray line), during a temperature increase from 30°C to 60°C (blue line), and at 60°C (red line).


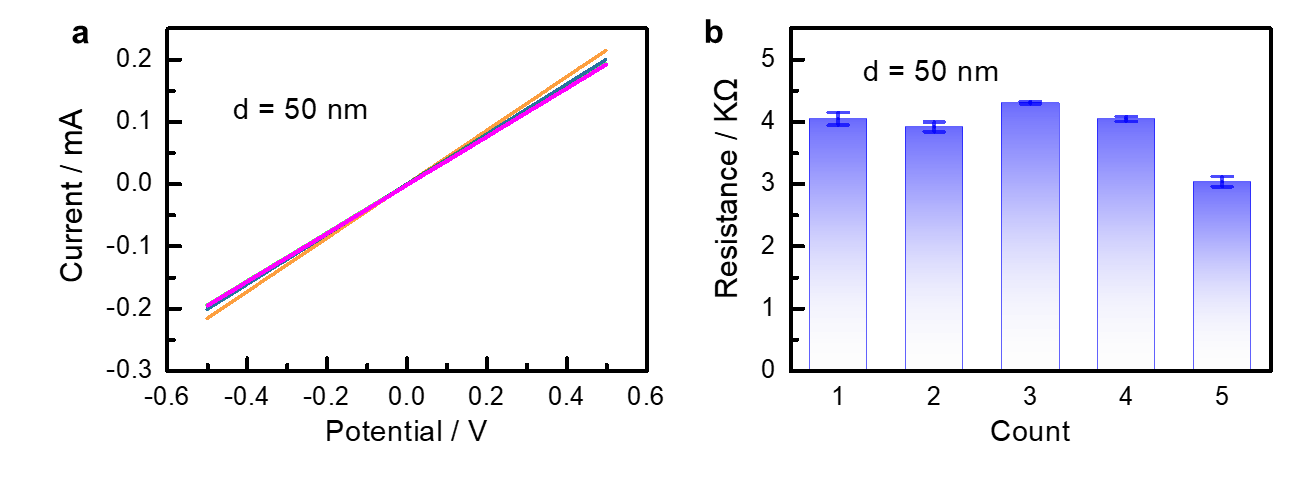


Fig. S4 **a** *I-V* curves of five individually prepared nanoprobes, each sputtered with a 50 nm layer of Pt and Ni. Scan rate = 100 mV s⁻¹. **b** Corresponding resistance of the prepared nanoprobes.


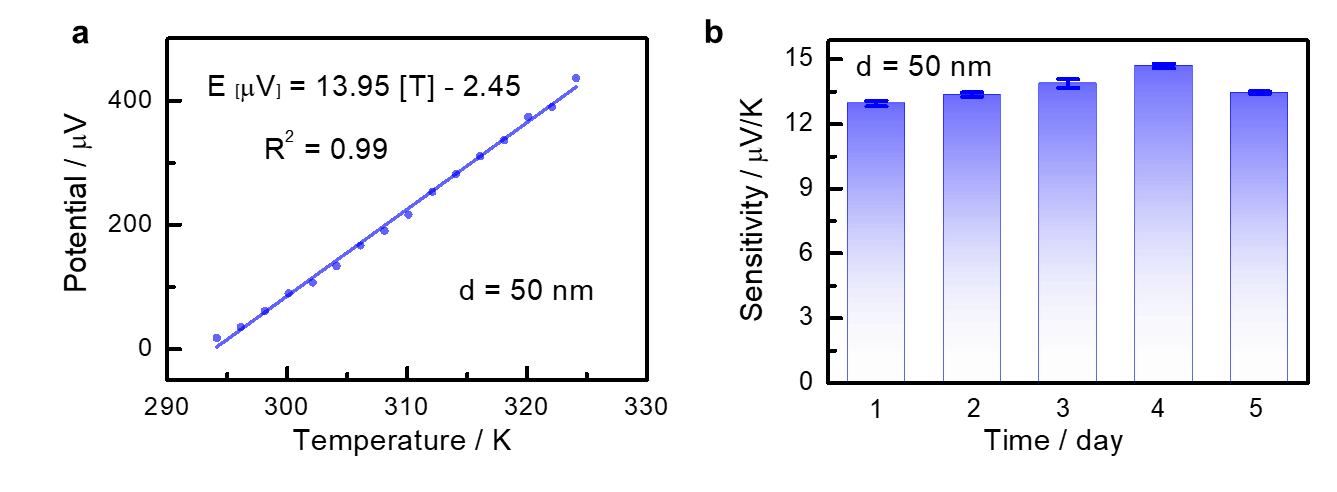


Fig. S5 **a** Linear dependence of voltage on temperature for the nanoprobe sputtered with a 50 nm layer of Pt and Ni, measured in 10 mM PBS. **b** Sensitivity of the nanoprobe over five days.


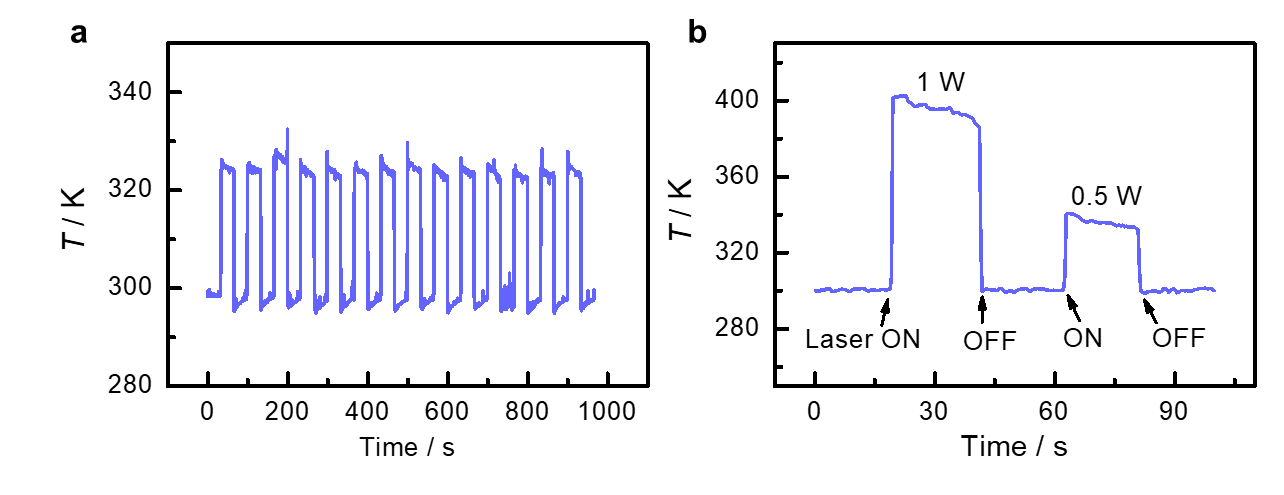


Fig. S6 **a** Temperature variation of the nanoprobe as the tip is periodically inserted into hot water (325 K) and retracted into air (298 K). **b** Temperature variation of the nanoprobe due to laser heating with powers of 1 W and 0.5 W, respectively. The slight decrease in measured voltage is due to the laser spot's position shifting relative to the nanoprobe tip over time, resulting in a decrease in the tip temperature.


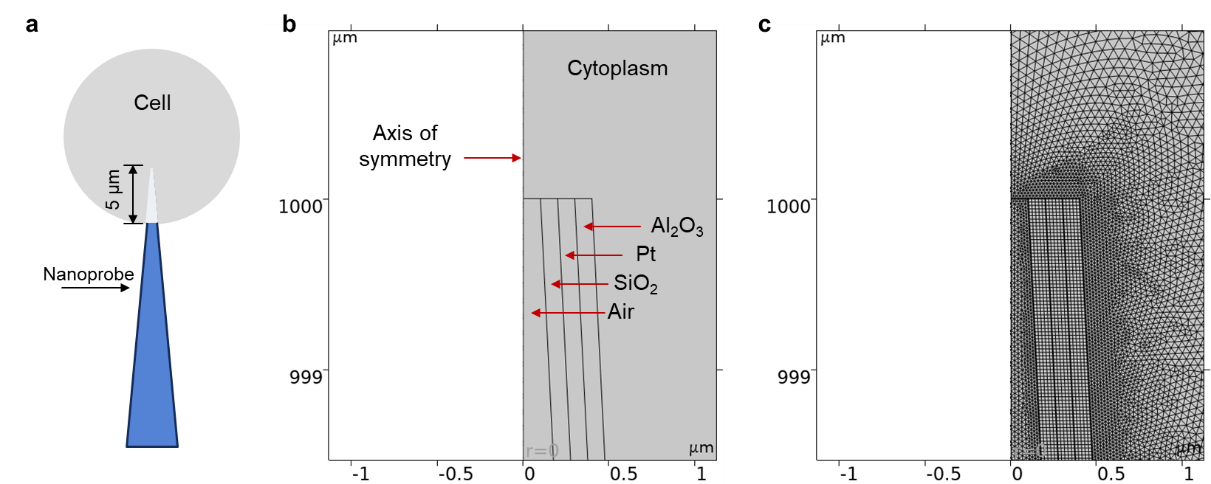


Fig. S7 **a** Geometry model used in the simulation, the length of the nanoprobe penetrating the cell is 5 µm. **b** Geometry of the model used in the simulation. **c** Mesh configuration of the model.


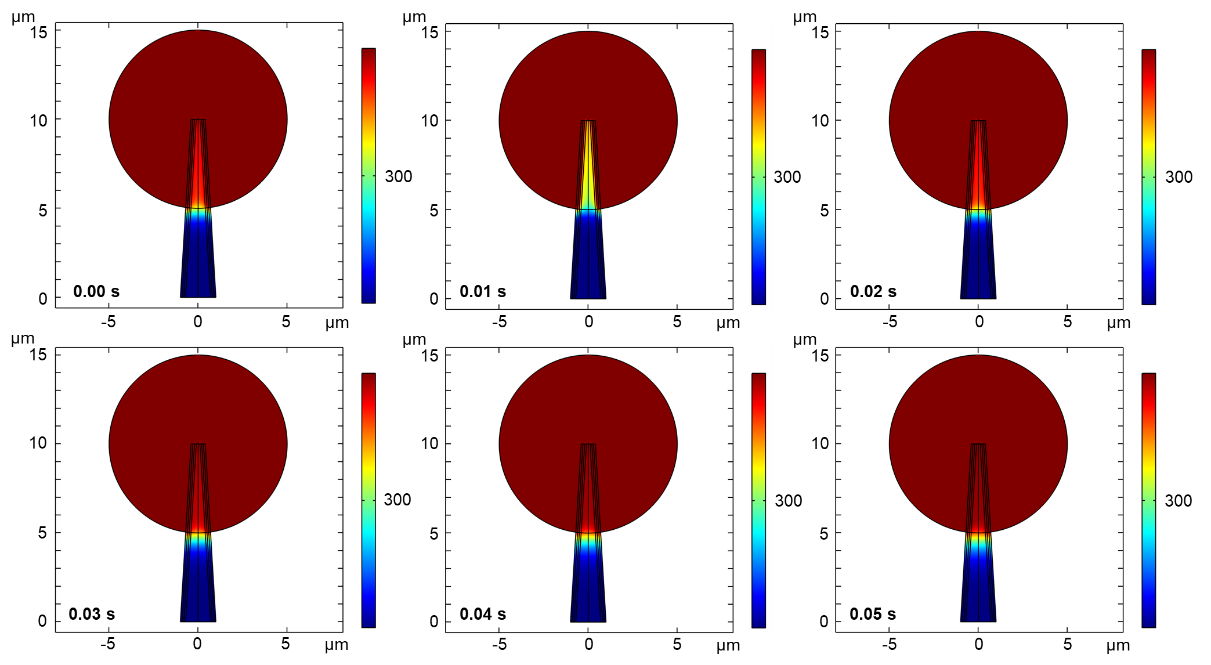


Fig. S8 Simulated response time of the nanoprobe when the cell temperature is 0.5 K higher than the nanoprobe.


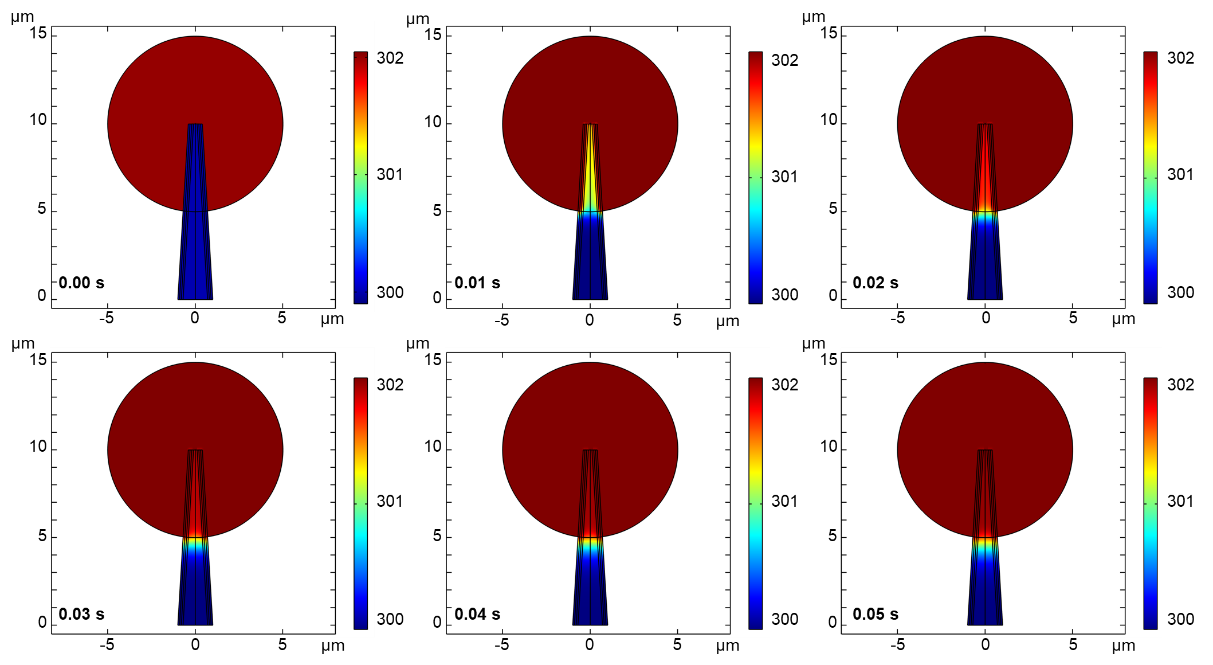


Fig. S9 Simulated response time of the nanoprobe when the cell temperature is 2 K higher than the nanoprobe.


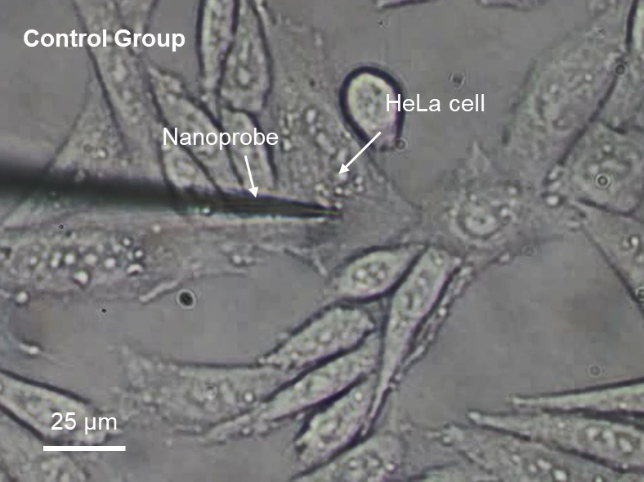


Fig. S10 Intracellular ROS measurement for HeLa cells that are not treated with Ti_3_C_2_@DOX (Control group).


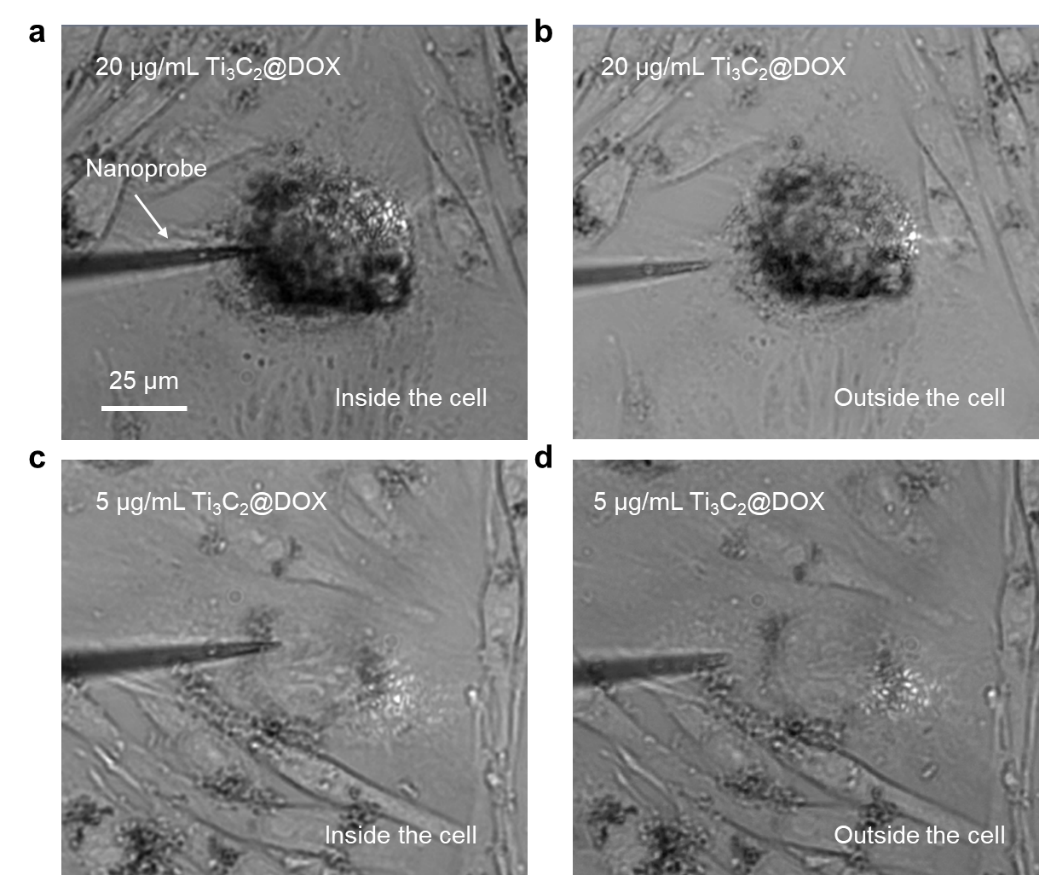


Fig. S11 **a** HeLa cells incubated with 20 µg/mL Ti_3_C_2_@DOX with the nanoprobe inserted into the cell. **b** HeLa cells incubated with 20 µg/mL Ti_3_C_2_@DOX with the nanoprobe near the cell. **c** HeLa cells incubated with 5 µg/mL Ti_3_C_2_@DOX with the nanoprobe inserted into the cell. **d** HeLa cells incubated with 5 µg/mL Ti_3_C_2_@DOX with the nanoprobe near the cell.
